# First Case of Acquired Hemophilia B in a Patient with HIV Infection: Case Report and Literature Review

**DOI:** 10.7759/cureus.4179

**Published:** 2019-03-05

**Authors:** Laura Páramo, Leonardo Jose Enciso Olivera, Ivan Noreña, María A Amaya, Juan C Santacruz

**Affiliations:** 1 Internal Medicine, University of La Sabana, Samaritan Hospital, Bogota, COL; 2 Hematoloty-Oncology, National Cancer Institute, Bogota, COL; 3 Infectious Diseases, Cardioinfantil Foundation, Bogota, COL

**Keywords:** hiv, acquired hemophilia

## Abstract

Acquired hemophilia B is a rare bleeding disorder in which circulating specific antibodies inhibit the coagulation factors. Usually, it is associated with autoimmune diseases, malignancy and some infections such as hepatitis B (HBV) and C (HCV) viruses. The association with the human immunodeficiency virus (HIV) is rare. We present the first case of hemophilia B acquired in a patient with HIV.

A 36-year-old woman with a history of HIV infection stage 3 acquired immunodeficiency syndrome (AIDS) (CDC 2014) and glomerulopathy secondary to HIV. She consulted for subacute non-dysenteric diarrhea. During her hospitalization, bacteremia by methicillin-sensitive Staphylococcus aureus (S.aureus) was documented, requiring hemodialysis catheter replacement with profuse bleeding during the procedure.

The coagulation tests showed prolonged activated partial thromboplastin time (aPTT), normal prothrombin time (PT), mixing test with Rosner index: 8.8%, negative lupus anticoagulant, and specific tests for normal factor VIII and XI. The activity of factor IX was <50% and the inhibitor was increased, which confirms the diagnosis of hemophilia B.

Acquired hemophilia B in a patient with HIV infection is not a frequent association; it should be suspected in the context of bleeding with prolonged aPTT.

## Introduction

Hemostasis is a complex mechanism which has evolved over time. It recognizes damage and at the same time activates the coagulation cascade in order to avoid massive blood loss following blood vessel injury. This process includes three steps: the first is the exposure of prothrombotic factors, followed by platelet plug formation, and, finally, activation of the coagulation cascade. There are physiological mechanisms which oppose clot formation, including blood flow which removes activated pro-coagulation factors, and endothelial nitric oxide and prostacyclin which inhibit platelet activation and aggregation. Proteins C and S, antithrombin and plasmin are responsible for degrading or inactivating components of the coagulation cascade [1].

Acquired coagulation inhibitors are specific circulating antibodies against the coagulation factors, principally IgG immunoglobulins of the IgG4 subclass [2-3]. These act by favoring the elimination or altering the function of the coagulation factor [4]. They generally inhibit factor VIII and the von Willebrand factor. However, they have been described against other coagulation factors, with limited reports in the literature [3]. The presence of acquired inhibitors against factor IX, or acquired hemophilia B, is a rare phenomenon [5] characterized by spontaneous bleeds of variable severity, especially manifested in postpartum hemorrhage [6-7]. Factor IX inhibition is mediated by IgG1 and IgG4 polyclonal antibodies [4].

Half of the causes of acquired hemophilia are frequently associated with conditions such as autoimmune diseases, lymphatic malignancies, medications, pregnancy, graft vs. host disease in allogeneic bone marrow transplants [8], and some infections, such as hepatitis B (HBV) and C (HCV) [9]; the other half are idiopathic. The association with human immunodeficiency virus (HIV) is rare, with four cases of acquired hemophilia A in HIV patients [10-12] and three cases in patients with HIV/HCV co-infection [9,13-14] reported in the literature. It is usually more common in the elderly and has a significant risk of mortality (15%-48%). The clinical course is variable, ranging from mild conditions to clinically significant, life-threatening bleeds [4]. Bleeding is limited mainly to the mucosa, skin or muscle; by contrast, bleeding caused by congenital hemophilia tends to mainly involve the joints [15].

The diagnostic approach in a clinically symptomatic patient begins with the measurement of clotting times; if these are abnormal, a mixing test should be performed. Treatment has two main objectives: control the bleeding and eliminate the inhibitor. The initial management consists of rest and avoiding surgical procedures and invasive diagnostic tests; in some cases, administration of antifibrinolytics may be effective [4]. In cases of life-threatening bleeding, recombinant factor VIIa may be used as a coagulation cascade bridge. Likewise, the elimination of anti-factor IX antibodies may be carried out using prednisolone monotherapy or together with cytotoxic agents such as cyclophosphamide. More recent studies have suggested the use of rituximab with, however, controversial results, in small populations and without reproducibility of their findings [16].

The purpose of this paper is to present the first reported case of acquired hemophilia B in a patient with HIV infection.

## Case presentation

A 36-year-old patient was admitted to the emergency room due to a two-week history of copious, non-dysenteric liquid stools (more than 10 per day), associated with fever of up to 39°C and chills. 

The patient was diagnosed in 2014 with HIV infection, stage 3 acquired immunodeficiency syndrome (AIDS) (CDC 2014), and was receiving antiretroviral treatment with darunavir 800 mg/day, ritonavir 100 mg/day and raltegravir 400 mg/12h, with poor compliance. Since 2017, she has undergone renal replacement therapy (three times per week) due to glomerulopathy secondary to HIV, and she also has chronic heart failure secondary to viral myocardiopathy. At the time, she had a viral load of 1,510,739 copies/mL, log 10 6.18 and, CD4 count of 9 cells/mm3.

During her hospital stay, abdominal and gastrointestinal sepsis were ruled out with no evidence of opportunistic infections at this level. Methicillin-sensitive Staphylococcus aureus (S.aureus) bacteremia was documented, with a transthoracic echocardiogram which did not reveal any vegetations but did show a severely affected systolic function. She was scheduled for removal and reinsertion of her hemodialysis catheter; however, a prolonged activated partial thromboplastin time (aPTT) was reported (55 sec), with a normal prothrombin time (PT), no clinical evidence up to that point of bleeding, and a persistently abnormal confirmation test. She underwent insertion of a new left jugular catheter with no immediate complications. A few hours after the procedure, she presented profuse and abundant bleeding at the catheter insertion site. A transfusion of six units of fresh frozen plasma and two units of packed red blood cells (PRBCs) was ordered. The following day, she required another transfusion of fresh frozen plasma due to persistent bleeding. Five days later, she had another episode of massive mucocutaneous bleeding which involved venipuncture sites, mucosas, and the gastrointestinal tract, with melena. Once again, a transfusion of six units of fresh frozen plasma was ordered. With a prolonged aPTT of up to 500 seconds and active bleeding, 12 units of cryoprecipitate were administered, along with 360 mg of tranexamic acid and 10 mg of vitamin K. Follow-up lab studies showed anemia, with a hemoglobin of 3.4 mg/dl, requiring the transfusion of two units of PRBCs.

The mixing test results showed a Rosner index of 8.8%, normal fibrinogen and fibrinogen degradation products, and normal prothrombin times (Table 1). However, the aPTT was >500 sec, with persistent bleeding and dependence on transfusions.

**Table 1:** Mixing test (March 13, 2018)

TABLE 1: Mixing test (March 13, 2018)			
Tube	Control plasma	Patient plasma	PTT
1	100%	0%	35.9 s
2	80%	20%	36.6 s
3	50%	50%	41.5 s
4	20%	80%	46.3 s
5	0%	100%	55.9 s
Incubation at 37ºC for 2 hours			
1	100%	0%	36.4 s
2	80%	20%	37.9 s
3	50%	50%	41.3 s
4	20%	80%	47.4 s
5	0%	100%	55.5 s
Rosner index: 8.8%			

The patient was seen by our department, and a possible diagnosis of acquired hemophilia was considered. A total of 24 mg of recombinant activated factor VII were ordered as a bypass therapy with satisfactory progress and decreased aPTT, without any new episodes of bleeding. The patient was discharged, but was readmitted a week later due to a new episode of profuse bleeding at the catheter insertion site following its manipulation in the renal unit. The bleeding improved after administration of factor VIIa, with hemodynamic instability and decreased hemoglobin, which required the transfusion of two units of PRBCs.

Factor VIII activity and factor VIII inhibitor measurements were reported as normal. However, factor IX activity measures were abnormal <50%, with an elevated IX inhibitor >0.5 Bethesda units, and a Rosner index <12% suggesting the presence of an inhibitor, with a normal thrombin time (Table 2). Therefore, acquired hemophilia B was confirmed. She had no recurrence of bleeding episodes, and a few days later the patient requested a voluntary discharge.

**Table 2:** Labs during hospitalization aPTT: activated partial thromboplastin time.

TABLE 2: Labs during hospitalization							
	28/02	05/03	08/03	12/03	16/03	20/03	Admission 30/03
Hemoglobin (g/dL)	6.5	6.3	8.2	8.7	9.3	6.9	9
Hematocrit (%)	21	19.8	25.2	27.7	27	22	24.9
Platelets (/uL)	118,000	122,000	206,000	150,000	109,000	82,000	99,000
aPTT (sec)	-	55.1	-	45	>500	45.8	76
Factor IX activity (%)	-	-	-	-	-	-	48%
Factor IX inhibitor (or Bethesda)	-	-	-	-	-	-	0.5
Factor VIII activity (%)	-	-	-	-	119%	-	-
Factor VIII inhibitor (or Bethesda)	-	-	-	-	0.05	-	-
Fibrinogen	-	-	-	-	225	-	174
Lupus anticoagulant	-	-	-	-	-	-	Negative
Ristocetin cofactor (%)	-	-	-	-	160	-	-
Thrombin time (s)	-	-	-	-	18	-	-
von Willebrand factor (%)	-	-	-	-	270	-	-

## Discussion

Although paradoxical, autoimmune disorders are not infrequent in the progression of HIV infection, especially in the early stages or during the immune reconstitution syndrome [17]. Numerous autoimmune diseases have been described in patients with HIV infection such as sarcoidosis, systemic lupus erythematosus, autoimmune hepatitis, Grave´s disease or vasculitis [17]. The hematological disorders described include antiphospholipid syndrome, primary immune thrombocytopenia, autoimmune hemolytic anemia [17], and acquired hemophilia, with seven cases of hemophilia A [9-20] and our case, the first report of acquired hemophilia B in an HIV infected patient.

The pathogenesis of acquired hemophilia in HIV patients is unknown. However, various mechanisms for the autoimmune phenomena in these patients have been proposed [10]. The B lymphocyte dysfunction caused by the virus appears to play a pivotal role, which is supported by the presence of hypergammaglobulinemia, circulating immune complexes and an elevated number of spontaneously proliferating B cells [18]. In addition, the molecular mimicry between HIV proteins and autoantigens could cause antibody cross reactivity and lead to the development of autoimmune diseases [19].

The presence of specific autoimmune diseases depends on the patient´s degree of immunosuppression [18]. In 2002, Zandman-Goddard and Shoenfeld proposed four stages of autoimmunity in HIV patients based on their CD4 count and AIDS manifestations [20]. Our patient was in stage III with a CD4 < 200 cells/mm3 and AIDS manifestations, where autoimmune diseases are uncommon.

In the reported cases of hemophilia A in HIV infected patients, the most frequent manifestations have been soft tissue bleeding, ecchymosis, and hematuria [10-14]. Cases of hemarthrosis [10] and abnormal uterine bleeding [12] have also been described. Our patient presented a clinical picture similar to that of the reported cases with bleeding due to catheters and ecchymosis, and she also had upper gastrointestinal bleeding.

Following the diagnostic algorithm approach [20], our patient had hemorrhagic symptoms, with no family or medical history of bleeding, a prolonged aPTT, and a normal PT. Therefore, a mixing test was performed which did not demonstrate factor deficiency, the presence of lupus anticoagulant was ruled out and the specific tests for factor VIII and XI were normal. However, factor IX activity was <50% and the inhibitor was elevated. Thus, she was diagnosed with acquired hemophilia B.

The goal of acquired hemophilia treatment should be hemostatic control and the elimination of inhibitors [10]. The first line of treatment for bleeding control are bypass agents which include recombinant activated factor VII (rFVIIa) and activated prothrombin complex concentrate [20]. Other options which increase factor VIII levels in acquired hemophilia A are desmopressin and factor VIII concentrate [20]. Our patient received rFVIIa during her first bleeding episode, with a satisfactory response.

In order to eradicate the inhibitors, the condition which predisposed their development must be controlled, which can lead to their rapid elimination. However, in most cases, immunosuppressants are required for their eradication [20]. The most frequently used agents include prednisone, cyclophosphamide, azathioprine, vincristine, cyclosporine and rituximab [20]. Of the reported cases, six received corticosteroids [10-14] alone or combined with another immunosuppressant such as rituximab, vincristine or cyclophosphamide [10]. Our patient did not receive specific treatment due to her advanced state of immunosuppression.

## Conclusions

Acquired hemophilia B in HIV infected patients is not a common disease; however, it should be suspected in the context of bleeding with a prolonged aPTT. The development of tools for the diagnosis and treatment of these patients is very important.
